# Active Aging: A Global Goal

**DOI:** 10.1155/2013/298012

**Published:** 2013-02-13

**Authors:** Rocío Fernández-Ballesteros, Jean Marie Robine, Alan Walker, Alex Kalache

**Affiliations:** ^1^Department of Psychobiology and Health, Autonomous University of Madrid, 28049 Madrid, Spain; ^2^French National Institute of Health and Medical Research, Paris, France; ^3^Sheffield University, South Yorkshire, UK; ^4^International Longevity Center, Brazil

Throughout the world, populations are growing older; although in developed countries population Aging started early in the XX century [1], less developed countries begun more recently. Therefore, it can be stated that population Aging is a global phenomenon. Population Aging must be considered as a success of the human race since it is the product of a long adaptation process, that is, we are coincident with that the increase in life expectancy has been determined by the development of lifelong education, biomedical advancements, socioeconomic progress, and the democratic political extension of these social developments, which in some countries has doubled life expectancy in under a century. 

Nevertheless, since individual Aging is associated with illness and functional loss, and disability-free life expectancy (DFLE) is significantly lower than life expectancy at birth (LE), this global demographic transformation, sometimes called “the silent revolution,” is considered by many as a threat in terms of public health and economic costs. But, life expectancy with disability (LEwD) shows a very broad variability among the world: while in some countries an individual born in 2002 can expect to have 10 years in poor health, in others countries this expectation is reduced to less than 7 years. Differences both in LE and in LEwD are expressing the extent to which there are inequalities in environmental conditions which, to a large extent, account for the variability in the ways populations are Aging. 

From a population perspective, the new paradigm of successful Aging, or Aging well, started in the eighties based on the compression of morbidity as a key concept for the development of this new paradigm in contrast with the common understanding that longevity necessarily increases morbidity [2, 3], or produced a dynamic equilibrium ([4], for a review see [5]). Latterly, authors noted how, since the 1950s, in selected countries, mortality after the age of 80 years has steadily fallen, they showed evidence that human senescence has been delayed by a decade strongly associated (from 1850) with behavioral and healthy “best practices” [6, 7].

Aging is not only a population phenomenon but also an individual reality and experience. Biogerontologists stated that while a 25% of the ways individuals age are accounted by genetics, it can be estimated that 75% are due to environmental conditions, including those behavioral events who select external conditions [8]. Therefore, at individual level, Aging is a long process across the individual life span governed not only by age and genes but by the interactions between socioenvironmental conditions with personal and behavioral events [9, 10]. Thus, at the individual level, Aging is not an at random phenomenon: the individual is an agent of his/her own Aging process, and the capacity for Aging well-healthy and active-comes, in a certain extent, from decisions taken by individuals themselves as well as his or her behavioral repertoires learnt across the life span. 

From an evidence-based point of view, it has been during the last decades of the XX century, with, the so-called “new paradigm” in the field of research on Aging and in a broad sense in the science of gerontology: a positive view (for a review see [11]). Pioneers in this new paradigm are authors from several gerontological disciplines, that is, from the fields of biomedicine and social sciences such as Fries and Crapo [3], Fries [12], Rowe and Kahn [13], or Baltes and Baltes [14]. This positive view of Aging adopted several verbal rubrics: “healthy” [15], “successful” [13, 14], “optimal” [16], “vital” [17], “productive” [18], “active” [19, 20], “positive” [21] or, simply, “aging well” [12] or “good life” [22]. It is important to emphasize that all these terms are taken by several authors interchangeably when they review the field (e.g., [23–25]); others try to establish differences between healthy Aging, active or successful Aging, and productive Aging [26, 27]. 

The worldwide phenomenon of active Aging also brought an acknowledgement by the United Nations (UN) of the many challenges regarding Aging and national development, issues concerning the sustainability of families and the ability of states and communities to provide for Aging population, that is, active Aging is placed as key concept. In April 2002, representatives from 159 nations met in Madrid to convene the *Second UN World Assembly on Aging*, two decades after the first assembly celebrated in Vienna in 1992. Although during the last three decades cross-sectional, longitudinal, and intervention studies on “Aging well” have been published, active Aging has been defined for the first time in 2002, by the WHO, in the booklet “*Active Aging. A policy framework*” as “the process of optimizing opportunities for health, participation and security, in order to enhance quality of life and wellbeing as people age.” The concept active Aging was adopted by *the United Nation Madrid II International Plan of Action on Aging*. Moreover, active Aging inspired policies at national, regional, and international actions among them the last one has been head by the European Union which declared* 2012 *the *European Year of Active Aging and Intergenerational Solidarity*. In sum, active Aging can be considered as *a global goal and as a political concept *[28] and it has even been converted into a *mantra* in Aging societies.

From a scientific perspective, *active Aging* can be considered as an umbrella concept embracing a semantic space in which healthy, successful, or productive Aging are strongly related. All these terms are considered as multidimensional and multilevel concepts and all of them are referring to a positive way of Aging or “Aging well,” and, as mentioned above, opening a new paradigm in gerontology, based on the delay of senescence, the compression of morbidity and mortality, the diversity of the ways of Aging, and the plasticity of human nature in front of enrichment circumstances [11, 27, 29]. 

Although there is not an empirical definition of active Aging commonly accepted, there is a certain consensus that it embraces a set of domains: low probability of illness and disability, high physical fitness, high cognitive functioning, positive mood and coping with stress, and being engaged with life (see [11, 13]). Those expert definitions are coincident with what lay older adults consider; thus, more than two-thirds of lay people from several countries and several continents understand active Aging as a set of personal ingredients such as “remaining in good health,” “feeling satisfied with life,” “having family members and friends who are there,” “adapting to changes related to Aging,” and “taking care of oneself” [30]. 

It must be emphasized that since there is not a commonly accepted definition of active Aging, studies looking for prevalence show a very confused panorama. From the cross-sectional and longitudinal studies of healthy or successful Aging reviewed by Peel et al. [25], results yielded a broad range of successful agers: from 12.7% (“survival, high level of functioning”) to 49% (“old age having little or no disability prior to death) and Depp and Jeste's [23] review yielded an even broader range of prevalence ranging from 0.2 to 97%. Fernández-Ballesteros and her group [31] through very broad differences between “simple” (93% “absence of support needed” to 27.4% “no illness reported” and “combined” outcomes (from 27,4% through 15.5%) and also between subjective (e.g., 80% “life satisfaction” through “MMSE” score higher than 28, 47%). The most accepted conclusion to these and other results is that a commonly accepted operational definition of active Aging is requested. 

Researchers distinguish between active Aging as an outcome of a lifelong process to its determinants or predictors. Thus, at the population level WHO posited 6 main determinants of active Aging: behavioral styles, personal biological and psychological conditions, health and social services, physical environment, and social and economic factors. Research searching for determinants of active Aging distinguish long-term determinants such as education, socioeconomic status, profession, life styles, health status, personality factors, or cognitive aptitudes [31]. During the last decades several experimental studies have been published with very promising evaluation studies and results from multidomain active Aging promotion programs (e.g., Active Aging South Australia), or programs promoting specific domains of active Aging such as physical activity (e.g., California Active Aging project), promotion of cognitive fitness (e.g., ACTIVE program), social participation, and others. Much more evaluation research must be conducted in order to tests active Aging good practices, training, projects, or programs. 

In spite of the fact that there are a theoretical corpus of knowledge, empirical cross-sectional and longitudinal, experimental research on active Aging, and social plans and policies for promoting active Aging, much more research results, debates, and discussions are required in order to make a step forward in this field. That is the main goal of this special issue on the 2012 European Year of Active Aging and Intergenerational Solidarity.

Ten papers are published in this special issue, by authors from around the world, contributing to some of the problematic issues we outline in our editorial and some of them enlighten with cross-cultural results on active Aging coming from several countries and regions. 

Regarding theoretical models of active Aging, C. Paúl et al. try to validate the population WHO 6 determinants factors of active Aging in a sample of Portuguese community-dwelling older adults in their contribution entitled “*Active Aging: an empirical approach to the WHO model*.” Performing a factorial equation modeling, they do not confirm the original model, but they arrived at a six-factor model where individual factors are explaining a 54% of the variance: health, psychological factors, cognitive performance, social relationships, biobehavioral components and personality. Much more research testing the WHO model (both outcome definition and posited determinants) from a multilevel population perspective is required.

In “*The theory and practice of active Aging*” J. F. Fries returns to the dynamic interaction of morbidity and mortality trends, the subject of his pioneering research more than three decades ago, and specifically to the erroneous assumption that morbidity would continue to develop at a specific age while mortality could be postponed continuously. Analyses of data from two controlled longitudinal studies of Aging, supported by the wider literature, suggest that exercise improves health in terms of both mortality and cumulative lifetime disability. Most importantly, this paper demonstrates that the absence of risk factors, such as lack of exercise, smoking, and overnormal body weight, is associated with a postponement of disability that significantly exceeds the postponement of mortality (6.7–9 years) and, therefore, a compression of morbidity closer to the age of death. 

Exploring the prevalence of active Aging based on Rowe and Kahn's model in a community dwelling sample of Western Mexico assessed through the SABE Protocol (which is being administered through Latin American countries by PAHO), E. D. Arias-Merino et al. are reporting their results in the paper “*Prevalence of successful aging in the elderly in Western Mexico*.” An average of 12.6% older adults were considered “aging well.” As in others studies, significant differences were found by age (lower percentage in those older), gender (women), education (lower education), and marital status (single). 

In the paper “*Social determinants of active aging: differences in mortality and the loss of healthy life between different income levels among older Japanese in the AGES cohort study*” H. Hirai et al. explore the relationship between income and loss of healthy years in a large sample of persons aged 65 or older in Japan. Within the Aichi Gerontological Evaluation Study (AGES), functionally independent elderly people have been followed during four years. The authors found that people with lower incomes were not only more likely to die than those with higher incomes but also more likely to report loss of healthy life years. This paper underlines the significant roles of social factors and social inequalities even in a rather egalitarian country. 

Another important contribution to this special issue comes from the very informative description of the CIS (former Soviet Union) countries made by A. Sidorenko and A. Zaidi from the European Center for Welfare Policy and Research (Vienna) in the paper “*Active Aging in CIS countries: semantics, challenges and responses*.” This highlights a region not very well known from an Aging and active Aging point of view. Coming from a period of financial instability and immersed in a accelerated processes of Aging, it is highly promising to learn that political actions, such as the 2012 European Year of Active Aging, are promoting health and independent living of older adults.

 From the Institute of Gerontology (University of Heidelberg, Germany), A. Kruse and E. Schmitt contribute to this special issue with the paper “*Generativity as a route to active Aging*.” After discussing the importance of active Aging from an individual as well as from a societal perspective as human capital, they focus on the psychological construct of generativity, reporting results from Mexico and Baltic countries and their cooperative research under the Dialogue Forum Project Funding, yielding interesting improvements of generativity in Belarus, Russia, and Ukraine by implementing and supporting local initiatives offering opportunities for intergenerational dialogue and complementing the results from A. Sidorenko and A. Zaidi and E. D. Arias-Merino et al.

 A theoretical article deals with a dynamic system model, the Janus model of development, “*On the dynamics of active aging*” by J. J. F. Schroots (Free University of Amsterdam, The Netherlands). The author provides very powerful theoretical and methodological tools for understanding the nature of development, based on the simplest possible set of underlying principles: the unitary lifespan trajectory with two complementary forces, growth and senescence, the peak capacity and peak time refer, respectively, to the impact of growth rate (peak capacity) and rate of senescence (peak time). Perhaps, most importantly, the validity of those principles is supported by simulating the empirical lifespan trajectories of functional capacity, intelligence, and mortality.

In their investigation of “*Mobility and active Aging in suburban environments: findings from in-depth interviews and person-based GPS tracking*,” E. Zeitler et al. use person-based GPS tracking to explore how suburban environments have an impact on older people's mobility and their use of different forms of transport. They found that suburban environments can create barriers to mobility which restrict the potential for activity in later life. Inaccessible public transport has the effect of increasing car dependence among older people. 

“*The right to move: a multidisciplinary lifespan conceptual framework*” is a serious call by the Health across Life Span work group of the University of Michigan's Society directed by T. C. Antonucci et al. proposing a proactive model to combat increasing inactivity associated with increasing obesity. This paper compliments that of J. F. Fries and details the benefits of physical activity from cells to culture through five intermediary organizing levels, such as family, community, and corporation. According to the authors, physical exercise is a powerful and low-cost solution to improve cognitive, emotional, and physical health and well-being. This paper demonstrates how and why all stakeholders have an interest in participating or contributing to such a move.

The paper “*Active aging promotion: results from the Vital Aging program*” by M. Caprara et al. (from several Spanish and Latin American Universities) describes a program promoting active Aging at the individual level. “Vital Aging” has been developed and tested in Spain as well as in several Latin American countries. The program targets individual determinants of active Aging, such as physical exercise, balanced nutrition, cognitively challenging activities, positive affect, and sense of mastery. The paper presents four evaluation studies corresponding to different formats of the proposed program, including e-formats. Limitations and futures steps are discussed. A large part of the paper is devoted to clarify the terminology used when aging is considered from a positive perspective. Thus, the authors describe a semantic network of aging well, including active, healthy, successful, productive, competent, vital, or optimal aging.

 Our gratitude is to all authors for their outstanding contributions.

*Rocío Fernández-Ballesteros* *Jean Marie Robine* *Alan Walker* *Alex Kalache*
